# Unravelling AYUSH providers’ perspectives on healthcare choices of people residing in urban areas of Puducherry, India- A concurrent mixed method study

**DOI:** 10.1016/j.jaim.2025.101180

**Published:** 2025-08-22

**Authors:** Anusha Seelamantula, Premarajan K C, Mahalakshmy Thulasingam

**Affiliations:** a JIPMER International School of Public Health (JISPH), Department of Preventive and Social Medicine, Jawaharlal Institute of Postgraduate Medical Education and Research (JIPMER), Puducherry, 605006, India; b Department of Preventive and Social Medicine, Jawaharlal Institute of Postgraduate Medical Education and Research (JIPMER), Puducherry, 605006, India

**Keywords:** AYUSH, Integration, Medical pluralism, Traditional, Complementary and alternative medicine, AYUSH healthcare providers

## Abstract

**Background:**

The rising popularity of alternative medicine positions AYUSH (Ayurveda, Yoga & Naturopathy, Unani, Siddha, and Homoeopathy) systems as valuable tools for providing affordable, culturally appropriate healthcare to low-middle-income countries. However, overcoming the challenges of scaling up these traditional systems is key to gaining broader acceptance of this promising healthcare approach.

**Objectives:**

To determine the prevalence and associated factors of AYUSH utilization. And, to explore the perceptions of AYUSH providers on factors supporting or hindering AYUSH adoption, and its integration with conventional medicine.

**Material and methods:**

A concurrent mixed-method study was employed between September and December 2023. Prevalence of AYUSH utilization was assessed via door-to-door survey using pre-tested questionnaire. Ten key informant interviews were conducted among AYUSH practitioners and analyzed using thematic analysis.

**Results:**

Prevalence of AYUSH utilization was 19.2% (95% CI:16.9% – 21.6%). A significant association among people with comorbidities (aPR: 1.5; 95% CI: 1.0–2.1; *P*-value<0.05) is observed. Qualitative analysis revealed that growing public interest and government support were key enablers, while limited scientific validation and workforce shortage were major barriers. From the health systems perspective, AYUSH providers spoke about the need for policy and governance reforms to create a more unified healthcare system.

**Conclusion:**

Despite some challenges, the findings suggest that AYUSH is increasingly favoured for its natural approach. A growing acceptance of integrative medicine highlights the need for cohesive care models and improved accessibility. The study emphasizes the importance of addressing state-specific needs and standardizing AYUSH practices to facilitate integration.

## 1 Introduction

The Alma-Ata Declaration 1978, introducing the concept of primary health care remains a pivotal moment in the global health history, advocating for an inclusive and accessible approach to health [1]. Following this, the Beijing Declaration of 2008 endorsed advocacy for integrating these systems into the national health system [2]. At the World Health Organization (WHO) Traditional Medicine Global Summit 2023, the Gujarat Declaration called for recognition, regulation, and scientific validation of traditional practices, while urging countries to create national policies that promote alternative medicine in a safe, effective, and evidence-based manner [3]. Modern medical systems in resource-limited settings, can often be expensive and require significant infrastructure, trained professionals, and long-term investments. On the other hand, AYUSH practices are cost-effective and less resource-intensive, making them important in providing affordable healthcare [4]. In many countries, particularly India and other parts of South Asia, traditional practices have been part of the culture and daily life for centuries. With the increasing recognition of alternative medicine in public health, the Ministry of AYUSH has been responsible for providing greater institutional support and enhancing standardization of these practices into the broader healthcare policy of India [5].

In 2017, the National Health Policy delivered a need to create protocols for integrating AYUSH into mainstream healthcare to improve access for a wide range of population [6]. Since the primary goal of these systems is to enhance everyday life, incorporating Ayush-based interventions in national health programs will also aid in optimizing daily life for overall well-being [7]. According to the National Health Profile 2021, there are 7032 Primary Health Centres (PHCs), 2793 Community Health Centres (CHCs), and 484 District Hospitals (DHs) co-located with AYUSH facilities providing an excellent opportunity for the public to utilize these systems [8].

Despite the trend of seeking multiple healthcare providers, use of AYUSH varies significantly [9]. The prevalence of AYUSH utilization in Indian populations was high but variable in different studies, highlighting the complex interplay of cultural, socioeconomic, geographical, and attitudinal factors within diverse populations [[10], [11], [12]]. Hence, there is a need to address this complexity by streamlining policies for an integrated approach. Over the years, streamlining AYUSH services into co-located public health facilities has unfolded in its own distinct way [13]. Studies have revealed challenges that impede the potential of AYUSH viz. lack of robust scientific evidence, limited workforce, lack of clear cross-referral protocols, and minimal logistical and infrastructural support [14,15].

While research has explored people's preferences for AYUSH, a substantial gap exists in learning the optimal integration of these practices with modern medicine [16]. There also remains ambiguity in the legal and professional standing of AYUSH systems, particularly regarding their scope of practice [17]. While AYUSH practitioners are trained under government-recognized institutions, the delineation of their roles in modern medical settings often leads to debates over their competency and the potential overlap with allopathic medicine [18]. Recent studies have focused on the need for clearer regulation, standardization of education, and greater collaboration with conventional medical practitioners [19]. The current study is expected to provide deeper insights into the challenges faced by AYUSH providers, including issues related to recognition, professional autonomy, and the public's perception of their practices. It could also address the growing demand for more integrated healthcare approaches, where AYUSH and allopathic systems can work synergistically for holistic patient care. This study's objectives were to estimate the proportion of utilization of AYUSH and determine the associated factors. Additionally, by exploring the perspectives of AYUSH providers, we aimed to identify specific barriers and opportunities to integration with modern medicine.

## 2 Methods

### 2.1 Study design

A concurrent mixed-methods design was employed from September to December 2023, to assess health behaviours in the general population while also gaining a deeper understanding of the experiences of AYUSH providers in their practice. The study was done in two phases: quantitative (cross-sectional analytical study) and qualitative (key-informant interviews). Ethical clearance was obtained from the Institutional Ethics Committee (No. JIP/IEC-OS/2023/074).

### 2.2 Study setting

The study was conducted in Puducherry, a Union Territory of India with a population of about 12.4 lakhs [20]. The National Family Health Survey 2019-20 reported a sex ratio of 1112 women per 1000 men, with a literacy rate of 89.7% in women and 93.8% in men [21]. There are not many studies that explored AYUSH prevalence in the regional population of Puducherry. The JIPMER Urban Health Center (JUHC) serves a population of about 10,000 people who have easy access to healthcare services. However, the use of alternative systems like AYUSH has not been studied in this area. For this reason, we chose JUHC as the focus of our study.

### 2.3 Study participants

People residing in the JUHC field practice areas were included in the quantitative part of the study, and none were excluded to capture the full diversity of individuals who use AYUSH therapies.

For the qualitative part, registered AYUSH practitioners working in a public or private health facility in Puducherry were involved. No traditional healers or quacks (unqualified doctors) were interviewed in the study.

### 2.4 Sample size and sampling technique

The sample size was calculated using OpenEpi v3.0, considering 17.7% prevalence of AYUSH utilization [10], at 5% level of significance and 5% absolute precision. With 1.5 design effect, and 5% non-response rate, the estimated sample size was 353. Using systematic random sampling, every 6th household was selected randomly. From the complete list of households, the first house was selected by a computer-generated random number followed by every 6th house subsequently proceeded towards right. All members of the selected household were assessed for AYUSH utilization only.

In the qualitative interviews, we intended to understand the perspectives about healthcare choices, barriers to treatment, and integration of AYUSH rather than the specifics of each discipline's treatment methods. So, a thematic saturation has been achieved after ten interviews. Participants were chosen through purposive sampling, targeting providers with a minimum of five years of experience and those actively engaged with patients in their daily practice.

### 2.5 Study tools and operational definitions

A pre-tested semi-structured questionnaire was developed to capture healthcare-seeking behaviour for AYUSH treatments, exploring various factors that influence an individual's choice of treatment. The questionnaire was pilot-tested on a small sample to identify any issues with question clarity, bias, or response patterns that may skew results. Utilization of AYUSH was defined as ‘participants having used one/more of the AYUSH systems in the last 6 months.’

For qualitative, a semi-structured interview guide was developed by literature review and was subsequently reviewed by authors PKC and MT experienced in qualitative research. The guide was pilot-tested among peers to improve the comprehensiveness of the questions. Topic guides (professional background and training, consulting patient demographics, workload, job satisfaction, challenges in practice, legal recognition, funding, criticisms, logistics, government support, interactions with allopathic doctors, opportunities for cross-disciplines, views on future of AYUSH) with relevant prompts were developed and used during the sessions.

### 2.6 Study procedure

In the quantitative part, participants were informed about the study purpose and their participation was purely voluntary with the option to withdraw at any time. Data confidentiality was ensured, with access limited to the authors. As no foreseeable or unforeseeable risks were anticipated, compensation was not provided. Informed written consent was obtained before administering the questionnaire. One responsible respondent (≥18 years) in each household who was available during the time of data acted as a proxy to provide information on AYUSH utilization for the entire household (totalling to 1094 individuals from 353 households). However, other attitudinal factors were assessed only for the responsible respondents (i.e. 353, one from each household).

For qualitative, the purpose of the key informant interviews (KIIs) was explained to the participants, consent was obtained, and the interviews were audio recorded with their permission. Privacy and confidentiality while interviewing was assured. The author AS with an MPH background, and well-trained in qualitative research methodologies carried out the interviews.

### 2.7 Data analysis

The data was entered into EpiCollect 5 and analyzed using Stata v14. Continuous variables were expressed with mean and standard deviation or median and interquartile range (IQR), based on data distribution and later categorized as proportions where necessary. Categorical variables were summarised as frequency and percentage with 95% confidence intervals. The association of sociodemographic variables with AYUSH utilization was assessed using Chi-square, estimating the prevalence ratio. Variables (*P* value < 0.2) were included in a binomial logistic regression model to estimate adjusted prevalence ratio (aPR) with 95% confidence interval. All the analysis was carried out at 5% level of significance, and a *P* value < 0.05 was considered statistically significant.

For qualitative analysis, all audio recordings were transcribed within three days of data collection to prevent loss of information. Each interview ranged in duration from 23 to 48 minutes. To ensure quality, field notes were randomly compared with the audio recordings. No repeat interviews have been required. A hybrid coding approach was used: codes were inductively generated through multiple readings of the transcripts and then systematically organized into pre-defined categories in a deductive manner by the author.

## 3 Results

The mean age of the respondents was 47 (SD = 15) years. To evaluate overall utilization, respondents were interviewed about their own and their family members’ use of AYUSH in the last 6 months, including the ailment and type of system preferred for treatment. Out of all the respondents and their family members (i.e. 1094 individuals), 210 (19.2%) had used AYUSH for various ailments. Among the users of AYUSH, Homoeopathy was the most preferred (31.1%), followed by Siddha (24.5%), Ayurveda (23.5%), and Yoga/Naturopathy (22.6%). AYUSH was primarily preferred for musculoskeletal problems (24.5%), respiratory troubles (18.3%), and other chronic diseases like hypertension (13.6%) and diabetes (8.4%).

**Fig. 1:**
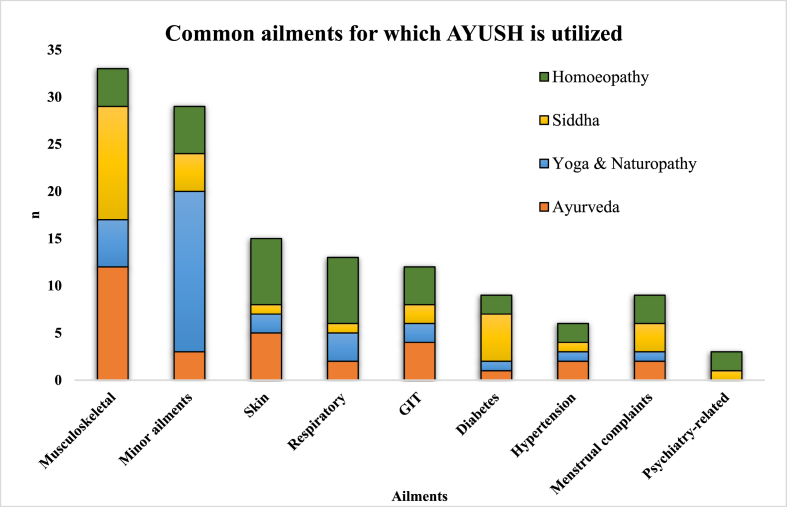
Type of ailments and the corresponding AYUSH system used (n = 101).

Since data was collected from one responsible respondent per household, the results presented pertain to them only. Table 1 presents the sociodemographic characteristics of the participants interviewed. Among these, 101 participants reported to be using AYUSH. Fig. 1 illustrates the type of AYUSH system used and the common ailments for which it was utilized.

**Table 1:** Socio-demographic characteristics of the responsible respondents from the selected households of urban Puducherry, India. (n = 353).

Variables	Frequency (n)	Percentage (%)
**Sex**		
Male	111	31.4
Female	242	68.6
**Religion**		
Hindu	315	89.2
Christian	35	9.9
Muslim	3	0.8
**Education of respondent**^a^		
No formal education	43	12.2
Primary (class I to IV)	21	5.9
Middle school (class V to VII)	48	13.6
Secondary (class VIII to X)	101	28.6
Higher Secondary (class XI to XII)	42	11.9
Graduation and above	98	27.8
**Occupation**		
Homemaker	147	41.6
Working	134	38.0
Not working	30	8.5
Retired	22	6.2
Student	20	5.7
**Marital status**		
Married	281	79.6
Single	41	11.6
Widow/Widower	25	7.1
Divorced/Separated	6	1.7
**Socioeconomic status**^b^		
I. Upper class (9098 and above)	88	24.9
II. Upper middle class (4550–9097)	116	32.9
III. Middle class (2729–4550)	62	17.6
IV. Lower middle class (1365–2728)	69	19.5
V. Lower class (1365 and below)	18	5.1
**Presence of comorbidities**^c^		
Yes	158	44.8
No	195	55.2
**Total**	**353**	**100**

a According to Indian Standard of Education Classification.

b Socio-economic status according to B.G. Prasad classification for October 2023.

c Hypertension, Diabetes Mellitus, Cardiovascular diseases, Chronic obstructive pulmonary diseases, Arthritis, and/or Renal disorders.

### 3.1 AYUSH treatment preferences

**Fig. 2:**
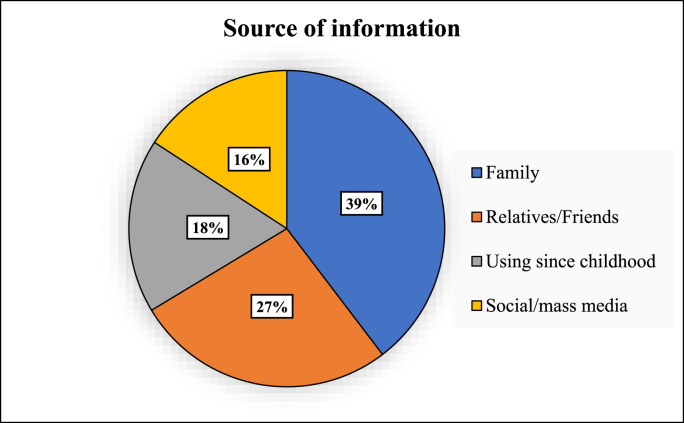
Source of information about AYUSH (n = 101).

Respondents' preference towards AYUSH and factors influencing the choice is shown in Table 2. About 55.4% of the participants used AYUSH as their main mode of treatment. 78.2% of participants reported visiting registered practitioners, suggesting a sense of security and trust in them. Only 5% directly approached a pharmacy for medication. Interestingly, more than half (57.4%) availed services from a private clinic while only 21.8% went to a government facility. Fig. 2 represents the participants’ source of information about AYUSH.

**Table 2:** Factors influencing the choice of AYUSH among the responsible respondents (n = 353).

Variable	n	%
**Reason for using AYUSH (**n **= 101)**		
Natural	42	41.6
No/Less side effects	27	26.7
Cost-effective	18	17.8
Well-known healthcare provider	14	13.9
**Reason for not preferring AYUSH (**n **= 252)**		
Lack of awareness	73	28.9
Long time to cure	49	19.4
Works for limited diseases only	41	16.2
Cannot use both allopathy and AYUSH at the same time	32	12.7
Presence of better allopathic alternative	28	11.2
Dietary restrictions	23	9.2
Restriction from family	6	2.4

### 3.2 Treatment perception

Among AYUSH users 75.2% used it for treatment purposes, 20.8% for promotion of health, and 4% for prevention of disease. While assessing the perception of treatment among those who use AYUSH, nearly half of them (44.6%) reported satisfaction. In terms of treatment outcome, almost 60.4% reported that the treatment was very effective while 3.9% stated that it was ineffective and hence discontinued treatment.

### 3.3 Service accessibility

About 51.5% of the participants travel up to 5–10 km to reach an AYUSH clinic and 16.8% travel for more than 10 km. Almost 82.2% reported that the drugs are easy to purchase, while only 5% reported that it was difficult. Over-the-counter and online purchasing is observed in 5.9% and 4% of participants respectively. The association of sociodemographic variables with the utilization of AYUSH is mentioned in Table 3.

**Table 3:** Association of sociodemographic variables with the utilization of AYUSH services by the responsible respondents (n = 353).

Sociodemographic variables	Total	Utilization of AYUSH		cPR (95% CI)	aPR (95% CI)	*P* value
		Yes n (%)	No n (%)			
Total	353	101(28.6)	252(71.4)			
**Age groups (in years)**						
18–25	29	5 (17.2)	24 (82.7)	ref	ref	–
26–44	126	39 (30.9)	87 (69.1)	1.7 (0.7–4.1)	1.4 (0.5–3.3)	0.50
45–59	117	31 (26.5)	86 (73.5)	1.5 (0.6–3.6)	1.1 (0.4–2.9)	0.85
≥60	81	26 (32.1)	55 (67.9)	1.8 (0.8–4.3)	1.1 (0.4–3.0)	0.77
**Sex**						
Female	242	72 (29.8)	170 (70.2)	1.2 (0.8–1.7)	–	–
Male	111	29 (26.1)	82 (73.9)	ref	–	–
**Education**						
No formal education	43	10 (23.3)	33 (76.7)	ref	–	–
Formal education	310	91 (29.4)	219 (70.6)	1.3 (0.7–2.2)	–	–
**Religion**						
Hindu	315	85 (27.0)	230 (73.0)	ref	ref	–
Non-Hindu	38	16 (42.1)	22 (57.8)	1.6 (1.0–2.4)	1.4 (0.9–2.1)	0.07
**Occupation**						
Not working	219	65 (29.6)	154 (70.3)	1.1 (0.8–1.6)	–	–
Working	134	36 (26.8)	98 (73.1)	ref	–	–
**Marital status**						
Married	281	86 (30.6)	195 (69.4)	1.5 (0.9–2.4)	1.3 (0.8–2.1)	0.33
Single	72	15 (20.8)	57 (79.1)	ref	ref	**-**
**Socio-economic status**						
Class I	104	36 (34.6)	68 (65.4)	1.3 (0.9–2.1)	1.4 (0.9–2.0)	0.06
Class II	105	29 (27.6)	76 (72.4)	1.1 (0.7–1.6)	1.2 (0.8–1.8)	0.38
Class III	144	36 (25.0)	108 (75.0)	ref	ref	**-**
**Presence of comorbidities**						
Yes	158	54 (34.2)	104 (65.8)	1.4 (1.0–1.9)	1.5 (1.0–2.1)	**0.04**
No	195	47 (24.1)	148 (75.9)	ref	ref	–

cPR- Crude Prevalence Ratio; aPR- Adjusted Prevalence Ratio; *p-*value<0.05 considered statistically significant.

### 3.4 Facilitator and Barriers to Utilisation of AYUSH Services

**Fig. 3:**
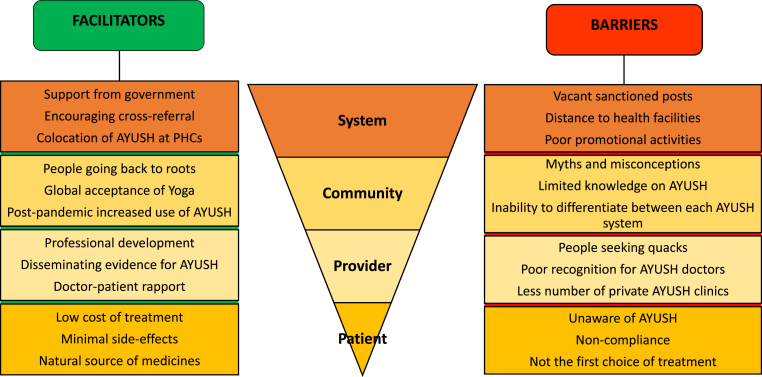
Conceptual framework of the perceived barriers and facilitators in the utilization of AYUSH from a provider's perspective (n = 10).

We interviewed ten key informant interviewees (four Siddha physicians, three Homeopathic physicians, two Ayurveda physicians, and one yoga practitioner), and the results obtained were represented under two themes (Table 4). A conceptual framework is illustrated in Fig. 3.

**Table 4:** Facilitators and barriers in the utilization of AYUSH.

Themes	Categories	Verbatim Quotes
**FACILITATORS**	Patient/Family level	*‘Our medicines are priced affordably, with low costs, making them accessible to everyone’* -Homoeopathic physician *‘Every system has its limitations, and ours is no exception. Although there may be some side effects, they are typically minor and can usually be handled by the body's natural processes without leading to any complications.’ -*Siddha physician
	Provider level	*‘We seize every opportunity that comes our way. For example, when invited to a school to give health education to children, we take that chance to explain how our system can help treat certain health conditions. As a result, when the need arises, we believe the children may inform their parents about Siddha. -*Siddha physician *‘Although we do receive some criticism, we also receive significant appreciation from allopathic practitioners. It's encouraging to see our efforts acknowledged by them. This recognition is likely a result of positive patient testimonials and the increasing body of evidence supporting our field of medicine.’ -*Siddha physician
	Community level	*‘Undoubtedly, Yoga has achieved global recognition. Nowadays, nearly all influencers and celebrities practice Yoga for maintaining a healthy body and mind. This trend is observed worldwide and presents a window of opportunity to promote other traditional systems of India.’ -*yoga practitioner ‘*Following the pandemic, there is a noticeable shift among the people towards enhancing immunity using AYUSH medications’-*Ayurveda physician
	System-level	*‘PHCs are co-located with both AYUSH and allopathic services, allowing individuals the flexibility to choose from different treatment options based on their preference’ -*Ayurveda physician
**BARRIERS**	Patient/Family level	*‘AYUSH is often suggested as a referral rather than being the primary choice for treatment; only a small number of individuals seek treatment directly from us initially.’* -Siddha physician
	Provider level	*‘When it comes to AYUSH matters, I'll have the final say, but I won't be involved in decision-making for other areas like health program implementations. While we're willing to assist, we don't receive much support’-*Ayurveda physician
	Community level	*‘VHNDs offer a valuable opportunity to educate individuals about the services available within the health system. Unfortunately, this platform is rarely utilized for raising awareness about AYUSH systems’*-Homoeopathic physician
	System-level	*‘Promotional activities are typically less frequent occurring primarily during special events such as Yoga Day or Ayurveda Day, when there is a greater focus on raising awareness and promoting these practices. Outside of these occasions, such activities tend to be limited’ -*yoga practitioner *‘AYUSH policy has remained unchanged since 2007, highlighting the need for a revised and updated version that aligns with the current patterns of seeking healthcare’*-Siddha physician

#### 3.4.1 Key facilitators

A mix of opportunities and challenges were mentioned by the AYUSH providers. They emphasized cost-effectiveness, as one of the primary reasons for people to choose these systems. They also stated, the increasing efforts by local government in promoting AYUSH, alongside independent attempts by providers, actively seizing opportunities to promote their services. Practitioners also highlighted growing public awareness on immunity and overall well-being. Government initiatives like integrating AYUSH into PHCs, and using platforms like VHNDs to raise awareness have become crucial in enhancing acceptance of AYUSH services.

#### 3.4.2 Key barriers

Providers mentioned lack of the public's understanding of the distinction between licensed AYUSH practitioners and quacks. An important observation made by a KI is that people often seek treatment from unqualified practitioners ignoring their credentials, as long as their symptoms improve. Accessibility to AYUSH clinics is hindered as they are typically located in providers' homes without designated facilities. AYUSH graduates often hesitate to open their own clinics due to concerns about the initial challenges and slow progress typical in the field, fearing their income might be insufficient. AYUSH doctors in facilities mentioned lack of decision-making and involvement in health programs.

### 3.5 Integration of AYUSH into conventional medicine

Adopting the WHO building blocks framework, the AYUSH providers’ experiences and challenges were organized as two themes (Table 5). When asked about how KIs perceive integration of AYUSH, a variety of statements were heard along the following lines: 'making AYUSH services accessible at primary care level', 'sensitizing ASHA workers to create awareness in the community about the assistance of AYUSH drugs in certain health conditions', 'creation of an integration among allopaths and AYUSH practitioners to provide appropriate care to the patients'.

**Table 5:** Experiences and Challenges of AYUSH providers in the integration of AYUSH.

WHO Building blocks	Verbatim quotes
**Health service delivery**	*I believe I should have the opportunity to propose to my fellow surgeons, “Let me try first, and if unsuccessful, surgery remains an option." Surgery isn't always the sole solution, and exploring alternatives can be beneficial –* Siddha physician *… the primary drawback is that AYUSH is rarely the initial treatment option for individuals. It is typically approached as a referral system, with patients turning to us only after conventional medicine has not yielded desired outcomes -* Homoeopathic physician
**Health workforce**	*The integration of allopathic and AYUSH doctors' perspectives can result in significant benefits for patients.* -Ayurveda physician *Our undergraduate students show a strong inclination towards research and overall improvement of system. I frequently advise my students, “Grow for yourself, the system will automatically grow”-* Siddha physician *Due to the limited availability of posts for AYUSH doctors, many new graduates opt for alternative career paths, leading to a brain drain from the system’* - Homoeopathy physician
**Health information system**	*We use the A-HMIS software, developed by MoA in 2018, which assigns a unique AYUSH ID to each patient within the portal, facilitating nationwide use across all five systems in India-* Siddha physician *While it was difficult to adapt, we eventually became accustomed to it, making our work much more streamlined-* Siddha physician
**Access to essential medicines and technologies**	*In Homeopathy, we typically prepare our medicines or procure them from reputable manufacturers known for their quality. We prioritize quality and are fortunate that our medicines have a prolonged shelf life.* - Homoeopathy physician *Numerous stores are advertising the sale of AYUSH drugs. While some of these claims may be genuine, many of these products do not adhere to the principles of our system and may yield different treatment outcomes, leading to people forming opinions based on their experiences with these products-* Homoeopathy physician
**Health financing**	*There has been a significant increase in the budget allocation for the MoA over the years, indicating the Government's keen interest in promoting the use of traditional medicines in the country -* Homoeopathy physician *The central government is allocating sufficient funds to support AYUSH systems. However, it is up to the state governments to ensure equal allocation of funds and resources to both AYUSH and allopathic systems.* -Siddha physician
**Leadership and governance**	*In my opinion, the State governments need to take the lead and keep these fragmented health systems in a satisfactory balance by maintaining coordination between the systems.* - Yoga practitioner *There is a need for updated revisions in the AYUSH policy. Many AYUSH practitioners lack awareness or show little interest in understanding the policy, focusing solely on treating patients who seek their services -*Siddha physician

A few respondents stated integration as follows:

‘Integration leads to drawing upon the strengths of all available health systems and promoting healthcare from below i.e. community-based right up to the higher health facilities’

One of the interviewees said that good coordination of both systems is needed.

‘A mutual strengthening of both the modern and AYUSH systems by a constant interaction between them so that people would receive a continuum of care according to their needs, allowing improvement of the effective role of AYUSH'

Overall, it is required to understand the complex mix of these hybrid (allopathic or traditional) health practices in the country to attain a striking balance in the health system.

In an interview, it was emphasized that Siddha medicine has broad applicability in addressing contemporary health challenges such as One Health. *‘One Health is not a new concept. It's an old wine served in a new glass for us. However, there is insufficient awareness about Siddha's potential contribution to this concept.’*

#### 3.5.1 Service delivery

The interviewees highlighted that integrating AYUSH and allopathic healthcare systems could significantly enhance service delivery if the benefits are acknowledged. However, they also pointed out significant barriers to integration, including patient resistance and their strong reliance on allopathic services, which could hinder the full utilization of integrated healthcare services.

#### 3.5.2 Health workforce

The providers stated that the government has acknowledged the need to integrate AYUSH with health and social services by promoting multi-disciplinary teams. This is by supporting educational institutions in cultivating AYUSH professionals and necessitates incorporating research findings into the system. On the other side, they also mentioned that many sanctioned posts for AYUSH providers in government facilities remain vacant, highlighting the need to fill these positions to enhance public access and prompt state governments to prioritize proper integration guidelines.

#### 3.5.3 Health information system

One interviewee highlighted a critical challenge: the need for clear communication and standardized data management. They suggested implementing consistent terminology and diagnostic codes across all healthcare systems. Additionally, it was perceived that absence of a uniform protocol for tracking AYUSH service utilization makes it difficult to accurately measure the true impact and reach of these practices.

#### 3.5.4 Access to essential medicines

The providers mentioned that the existence of a dedicated ministry has facilitated the seamless supply of drugs from approved manufacturing companies. The KIs showed no barrier in indenting drugs for smooth delivery of services. However, the gap lies at the consumer level, where individuals sometimes lack immediate access to purchase drugs from authorized pharmacies.

#### 3.5.5 Financing

An interviewee stated the country's increasing budget on AYUSH for research and wider service delivery. A proper alignment between the financial partners of the State and the Centre is a felt need. Transparency in fund utilization is also identified as a barrier to implementing integrated AYUSH services.

#### 3.5.6 Leadership/governance

There is a lack of clarity regarding AYUSH policy objectives for proper mainstreaming. The limited involvement of AYUSH practitioners in the decision-making processes within healthcare facilities appears to hinder their integration, restricting the full utilization of their potential.

## 4 Discussion

Our study has shown that people with chronic comorbidities, higher socio-economic status, and older age have increased utilization of AYUSH. The outlook of AYUSH providers on medical pluralism, identified that efforts must be focused on awareness, evidence-based practice, and quality assurance of drugs. The study evaluated multifaceted opportunities within each block of health system, encompassing policy support to maximize the potential of AYUSH.

### 4.1 Utilization of AYUSH

Almost 19.2% of the participants reported using AYUSH for themselves or their family members. In India, wide variations in AYUSH usage ranging from 6.9% to 65% are found [11,22]. In a study by Pengpid and Peltzer from a nationally representative cross-sectional household survey of the National Sample Survey Organization (NSSO), a contrasting prevalence was observed in Uttar Pradesh (18.7%), Maharashtra (13.8%), Bihar (8.1%), Kerala (7.7%) and West Bengal (6.9%) [23]. Similarly, Priya and Shweta surveyed households from 18 states of India with utilization ranging from 30 to 60% [24]. In another study conducted by Singh P et al., 14% availed treatment from ISM and Homoeopathy [4]. WHO SAGE survey conducted in six middle-income countries has shown that 11.7% have frequently visited traditional healers as a frequent source of treatment [25]. This differences in proportions might be due to variations in recall period, cultural viewpoints, unit of study population, or sole focus of only one system in analysis.

We also found the dual use of AYUSH with allopathy (38.6%) noteworthy, pointing to a growing acceptance of integrative medicine. This underscores the need for collaborative care models, such as the National Institute of Mental Health and Neurosciences (NIMHANS) Model of System Integration of Medicine, which shifts from the traditional ‘cafeteria approach’ at co-located health facilities to a more cohesive Integrative Approach [26]. Similarly, only 21.8% of individuals accessed services from government AYUSH facilities, indicating potential challenges related to infrastructure, staffing, or a lack of public awareness about the availability of these services. Additionally, a significant group faced accessibility challenges, emphasizing the need for more AYUSH clinics, mobile health units, or telemedicine options to improve service reach.

Our study has also revealed about 32.1% utilization among elderly (≥60 years) showing a higher prevalence (aPR 1.1, 95% CI 0.4–3.0, *p* = 0.77) compared to younger age groups, with 26.7% citing it due to lesser side effects. A similar study by Srinivasan et al. found that 21.2% used alternative therapies due to negligible side effects [27]. An interviewee stated that there may be some, often minimal side effects in AYUSH treatments. Hence, establishing pharmacovigilance wings can help monitor, detect, and assess adverse effects, ensuring patient safety and improving care quality. A higher footfall for non-communicable disease (NCD) and geriatric OPDs was also stated, signifying that people perceive AYUSH as highly focused on promoting lifestyle modifications. Another observation was that people belonging to upper socioeconomic classes showed higher utilization (aPR 1.4, 95% CI 0.9–2.0, *p*=0.06), likely due to better awareness and preference towards preventive health measures. Additionally, family (39%) and friends/relatives (27%) were observed to be major motivators to the use of AYUSH, which is similar to Vandana et al. findings where about 43% were advised by family and 27% by friends [28]. The AYUSH providers have also reported that most patients come to their clinics when referred by one of their relatives who has been satisfied with the treatment.

Previous literature shows that some individuals choose AYUSH therapies directly, while others try allopathic medicine, find it ineffective, and then explore traditional AYUSH care [29]. The use of AYUSH therapies can alleviate pressure on overburdened healthcare systems by providing alternative treatment options that are both effective and sustainable. The interviews indicated that integrating AYUSH and allopathic treatments can significantly reduce the dosage of modern medications while maintaining effectiveness.

A deep and nuanced view of the current state of AYUSH is perceived in the study. These findings highlight the need to increase awareness, standardize methodologies, enhance accessibility, and establish feedback mechanisms through community outreach and educational campaigns targeting healthcare professionals, policymakers, and the general public to improve AYUSH services. Promoting these systems which are deeply rooted can also enhance community engagement and health literacy, empowering individuals to take control of their own health in culturally meaningful ways. The visibility of government support can also help reduce stigma or skepticism that exist around traditional medicine. While advocacy may raise awareness, it needs to be coupled with credible information and public health campaigns that provide clear picture of the benefits of AYUSH.

### 4.2 AYUSH Integration

In the qualitative analysis, we found that the opinions between AYUSH providers have aligned. While AYUSH departments are being integrated into institutes like, All India Institute of Medical Sciences (AIIMS), to enhance healthcare delivery, providers noted inadequate infrastructure of AYUSH clinics and lack of opportunities as potential brain drain to new graduates. This could be improved by developing clear standards for AYUSH education and practice which in turn will improve the credibility of the professionals. Another noteworthy aspect is the increase in budget for MoA over the years. However, there is ambiguity regarding the state-wise budget utilization. Hence, state-specific needs within AYUSH sector are required in order to negotiate with the Centre. It is understood that a new integrated policy is needed to guide both AYUSH and allopathic providers amidst fragmented health systems. Programs like Yoga Day have significantly impacted many areas, especially the school children. After integration into the school curriculum, a study by Manjunath N reported that it helped children in fitness, stress management, and academic performance [30]. A KI also suggested integrating Yoga into schools and workplaces to improve mental health. These insights can be adopted to advance the role of AYUSH within broader health system.

### 4.3 Recent key initiatives

Each system of AYUSH claims the potential to treat a broad spectrum of diseases, with various national institutions working to disseminate this information [15]. AIIMS New Delhi is researching geriatric care and post-CABG rehabilitation in the community, where AYUSH therapies may offer complementary benefits, such as improving mobility, reducing stress, and enhancing cardiovascular health. Similarly, AIIMS Bhopal and DMIMS Wardha have initiated research on sickle-cell disease, and a multicentric study on rheumatoid arthritis is ongoing [31]. Ayurtech IIT Jodhpur is creating an AI-driven framework for risk stratification and early health interventions [32]. As AYUSH treatments gain increasing attention in research and clinical practice, there is also a pressing need for a regulatory framework that ensures standardization, quality control, and safety of AYUSH medicines.

### 4.4 Strengths and limitations

The study has several strengths. First, it examined AYUSH utilization at the household level, allowing for an in-depth understanding of intra-family differences in healthcare choices. The qualitative component provided valuable insights into the practical challenges and perceptions of AYUSH providers. Additionally, being a community-based study, it captured a broader and more diverse sample, enhancing its external validity. Including all AYUSH systems offered a holistic view, and the six-month recall period has minimized recall bias. Nonetheless, there were some limitations. The field practice areas of JUHC have relatively easy access to health services, which may have introduced reporting bias, as participants could have overrepresented the use of accessible healthcare options. Additionally, while the study focused on AYUSH utilization patterns, it did not include in-depth interviews with beneficiaries, which could have provided richer insights into patients' personal experiences and decision-making processes. Furthermore, the study did not explore potential differences in AYUSH preferences between healthy individuals and those with specific health conditions, which could have offered better understanding of choice based on health status.

## 5 Recommendations

There are few misperceptions and a lack of awareness about AYUSH. Providing credible information from reliable sources is crucial, as evidence indicates a tendency for people to consult quacks rather than licensed AYUSH professionals. Introduction of these systems in education at younger age will bring about a cumulative effort in sensitization. Many respondents have reported that they use AYUSH medicines as they are cost-effective. However, most participants accessed the services at private clinics, so it is important to improve the availability of AYUSH clinics. A quality check and implementation of stricter regulations and licensing procedures is required on AYUSH to promote ethical practices and maintain standards.

## 6 Conclusion

It was noticed that nearly one-fifth of the participants were using AYUSH systems in the study setting. Additionally, the knowledge about AYUSH systems has not yet invaded the population despite the increased advocacy from the government. We perceived from the interviews that, AYUSH treatment if used along with allopathic medications can be beneficial to the patients as it can help with a better recovery. Since it was observed in the study that most elders prefer AYUSH systems, it can play a significant role in meeting the healthcare needs of the ever-increasing geriatric population of India. It is also perceived that these medicines are safer and gentler on their bodies when compared to the heavy doses of modern medicine. Better promotional activities with easy accessibility of AYUSH clinics to the general population may serve better in the long run for increasing the utilization of AYUSH. Overall, integrating AYUSH with conventional healthcare by understanding the healthcare-seeking behaviour of the people caters to their diverse needs and preferences.

## Author contributions

AS- investigation, validation, data curation, formal analysis, writing of original draft. PKC and MT-supervision and project administration. All authors have contributed to the conceptualisation, review and editing of the manuscript. They have all read and approved of the final version.

## Declaration of generative AI in scientific writing

The authors declare that generative AI and AI-assisted technologies were not utilized in the writing process.

## Funding sources

The study was supported by the JIPMER Intramural Research Fund Committee.

## Conflict of interest

The authors declare that they have no known competing financial interests or personal relationships that could have appeared to influence the work reported in this paper.

## References

[1] WHO and UNICEF (2018).

[2] Nambiar D., Narayan V.V., Josyula L.K., Porter J.D.H., Sathyanarayana T.N., Sheikh K. (2014). Experiences and meanings of integration of TCAM (Traditional, Complementary and Alternative Medical) providers in three Indian states: results from a cross-sectional, qualitative implementation research study. BMJ Open.

[3] Editorial S. (2023). WHO traditional medicine global Summit 2023 meeting report: Gujarat declaration. J Ayurveda Integr Med.

[4] Singh P., Yadav R.J., Pandey A. (2005). Utilization of indigenous systems of medicine & homoeopathy in India. Indian J Med Res.

[5] Singh A. (2016). National Ayush mission- operational guidelines. Ministry of AYUSH.

[6] Ministry of Health and Family Welfare (2017).

[7] Gupta P., Karthik K., Sahu R., Shrikrishna R., Mahapatra A. (2023). Public health initiatives and Ayush: projects to policy. Int J Ayurveda Res.

[8] Kumar S., Gopal K.M., Choudhary A., Soman A., Namburi U.R. (2023). Advancing the one health approach through integration of Ayush systems: opportunities and way forward. J Family Med Prim Care.

[9] Mohanty P., Kishore J., Acharya G.C., Mohanty I., Patnaik L., Bhowmik B. (2024). Utilization of Ayurveda, yoga, Naturopathy, Unani, Siddha, and homoeopathy (AYUSH) practitioners' services among older adults: results from the longitudinal aging study in India. Cureus.

[10] Kumar S., Shetty A., Bhat S., Prabhu S., Dsouza O., Krishna Kn (2021). Complementary medicine utilization and practices of self-medication in the field practice areas of a medical college of district Dakshina Kannada, Karnataka, India. BLDE University Journal of Health Sciences.

[11] Rudra S., Kalra A., Kumar A., Joe W. (2017). Utilization of alternative systems of medicine as health care services in India: evidence on AYUSH care from NSS 2014. PLoS One.

[12] Kshirsagar V., Tiwari S., Thingore C., Limaye D. (2020). The practice, attitude, and knowledge of complementary and alternative medicine in Mumbai, India. Int J Community Med Public Health.

[13] Samal J. (2015). Role of AYUSH workforce, therapeutics, and principles in health care delivery with special reference to National Rural Health Mission. AYU (An International Quarterly Journal of Research in Ayurveda).

[14] Thyagarajan S. (2023). Integration of Ayush within national health care systems: challenges and the way forward. Journal of Research in Ayurvedic Sciences.

[15] Javed D., Dixit A.K., Anwar S., Giri N. (2024). Exploring the integration of AYUSH systems with modern medicine: benefits, challenges, areas, and recommendations for future research and action. J Prim Care Spec..

[16] Shrivastava S.R.B., Shrivastava P.S., Ramasamy J. (2015). Mainstreaming of Ayurveda, yoga, Naturopathy, Unani, Siddha, and homeopathy with the health care delivery system in India. J Tradit Complement Med.

[17] Josyula K.L., Sheikh K., Nambiar D., Narayan V.V., Sathyanarayana T.N., Porter J.D.H. (2016). “Getting the water-carrier to light the lamps”: discrepant role perceptions of traditional, complementary, and alternative medical practitioners in government health facilities in India. Soc Sci Med.

[18] Lakshmi J. (2012). Less equal than others? Experiences of AYUSH medical officers in primary health centres in Andhra Pradesh. Indian J Med Ethics.

[19] Singhal S., Roy V. (2018). Awareness, practice and views about integrating AYUSH in allopathic curriculum of allopathic doctors and interns in a tertiary care teaching hospital in New Delhi, India. J Integr Med.

[20] Government of India (GOI) (2011).

[21] International Institute for Population Sciences (2022).

[22] Janak Yadav R., Yadav J., Siddique N., Pandey A. (2015). Knowledge and utilization of Indian system of medicine in the state of Assam. Indian J Comm Health.

[23] Pengpid S., Peltzer K. (2021). Utilization of complementary and traditional medicine practitioners among middle-aged and older adults in India: results of a national survey in 2017–2018. BMC Complement Med Ther.

[24] Priya R., Shweta A.S. (2010).

[25] Oyebode O., Kandala N.B., Chilton P.J., Lilford R.J. (2016). Use of traditional medicine in middle-income countries: a WHO-SAGE study. Health Policy Plan.

[26] Bhargav H., Holla B., Ramakrishna K., Shivakumar V., Gokulakrishnan K., Varambally S. (2022). Yoga and integrative healthcare: lessons from the national institute of mental health and neurosciences (NIMHANS) in India. Int J Yoga.

[27] Srinivasan R., Sugumar V.R. (2017). Spread of traditional medicines in India: results of national sample survey organization's perception survey on use of AYUSH. J Evid Based Complementary Altern Med.

[28] Roy V., Gupta M., Ghosh R.K. (2015). Perception, attitude and usage of complementary and alternative medicine among doctors and patients in a tertiary care hospital in India. Indian J Pharmacol.

[29] Shivanand B.S., Tushara M., Unnikrishnan P.M., Krishnamurthy J. (2022). Do integrative approaches to health contribute to self-reliance in primary healthcare? reflections from a community case study in Kerala, India. Health Psychol Behav Med.

[30] Patwardhan A.R. (2017). Aligning yoga with its evolving role in health care: comments on yoga practice, policy, research. J Prim Care Community Health.

[31] Ministry of AYUSH (2024).

[32] Chaudhari M.A., Jangid S.K. (2024). Exploring NEP 2020: Anticipations, Challenges and Actualities.

