# Supplementary material for: Unravelling AYUSH providers’ perspectives on healthcare choices of people residing in urban areas of Puducherry, India- A concurrent mixed method study
Source: J Ayurveda Integr Med. 2025 Aug 22;16(5):101180. doi: 10.1016/j.jaim.2025.101180 (PMC12398260; doi:10.1016/j.jaim.2025.101180)
Supplement: Multimedia component 1 [file mmc1.docx]

**(Table 6 submitted as supplementary material)**

**Table 6: Joint Display of Quantitative and Qualitative Results:**

| **Overarching themes** | **Quantitative results** | **Qualitative results** | **Meta inference** |
| --- | --- | --- | --- |
| Potential benefits of Ayush | Around 39% of households have at least one person using AYUSH. | AYUSH providers reported that despite government advocacy, public awareness remains low. The fragmented knowledge needs to be addressed to increase utilization rates.  **Supporting quote:**  *‘Recently government is taking initiatives on AYUSH. Now, at least* ***one or the other in a family uses*** *any of the AYUSH systems’* *-* Siddha physician | **Discordant:** Although nearly two-fifths of the households utilized any one of the AYUSH systems, the providers perceive that the awareness among people is still low. |
| Empowering patients through health education | In total, 57.4% of participants availed services from a private clinic. | People perceive AYUSH drugs as having fewer side effects, which drives them to visit a clinic even if it is private.  **Supporting quote:**  *‘Most AYUSH clinics are* ***private****, and only a few states have active AYUSH services in the PHCs’-* Homoeopathy physician | **Confirmed:** The general public receiving care from private clinics is consistent with the experiences stated by the provider |
| Improved accessibility to reduce travel burden | About 17.8% of AYUSH users travel distances above 10 kilometers to access services. | According to the key informants, although there are clinics in patients' neighborhoods, many are unaware of them because most AYUSH clinics are operated from providers' homes’  **Supporting quote:**  *‘When patients mention* ***challenges with follow-up*** *due to distance, we facilitate referrals to nearby clinics for their convenience’*- Ayurveda physician | **Expanded:** Providers believe referring patients to nearby clinics can improve treatment adherence and follow-up care, reducing the travel burden. |
| Addressing the myths and misconceptions | A significant number of respondents (28.9%) indicated lack of awareness regarding the efficacy of these medicines.  Additionally, 12.7% of respondents felt that AYUSH and allopathic medications cannot be used concurrently. | Misconceptions exist among the public about AYUSH systems. However, AYUSH providers emphasize that their treatments complement allopathy and can lead to faster recovery.  **Supporting quotes:**  *‘Some patients say* ***there are better allopathic alternatives*** *and that’s why they did not choose to come to our clinic until now, but* ***there*** ***is also an AYUSH alternative.*** *Every system has its own limitations that doesn’t mean ours is not effective in treating ailments.’-* Siddha physician | **Expanded:** AYUSH providers stated that integrative treatment i.e. along with allopathy, could be beneficial for the patient in better recovery. |
